# Partial hydrogenation of soybean oil over a Pd/bentonite catalyst

**DOI:** 10.1039/d5ra01198a

**Published:** 2025-05-02

**Authors:** Karina Herrera-Guzmán, Sheila Amellalli Francisco-Bustos, Eréndira Torales, Jesús Alejandro Peña-Jiménez, Rubén Gaviño, Eréndira García-Ríos, Oscar Abelardo Ramírez-Marroquín, José A. Morales-Serna, Jorge Cárdenas

**Affiliations:** a Instituto de Química, Universidad Nacional Autónoma de México, Circuito Exterior, Ciudad Universitaria Ciudad de México 04510 Mexico rjcp@unam.mx; b Colegio de Ciencias y Humanidades-Azcapotzalco, Universidad Nacional Autónoma de México Av. Aquiles Serdán Ciudad de México 02020 Mexico; c Centro de Investigaciones Científicas, Instituto de Química Aplicada, Universidad del Papaloapan Tuxtepec Oaxaca 68301 Mexico joseantonio.moralesserna@gmail.com

## Abstract

In the chemical industry, hydrogenation reactions play a significant role by saturating double bonds in vegetable oils. While most selective hydrogenation processes are carried out at high temperatures (120–180 °C), the development of catalytic systems for partial hydrogenation at low temperatures has been scarcely studied. In this context, we investigated a catalyst obtained by incipient wetness impregnating a Pd(ii) salt onto bentonite, following its reduction to Pd(0). Under these conditions, a metal loading of 1.4% was achieved. At 550 psi of H_2_, we proved the catalyst to be excellent for the partial hydrogenation of soybean oil at 25 °C and 70 °C, achieving iodine values (IV) of 76 and 71, respectively. These results are remarkable, particularly when considering the hydrogenation product as a potential feedstock.

## Abstract 2

Development of supported catalyst for the partial hydrogenation of vegetable oil under mild conditions.

## Graphical abstract

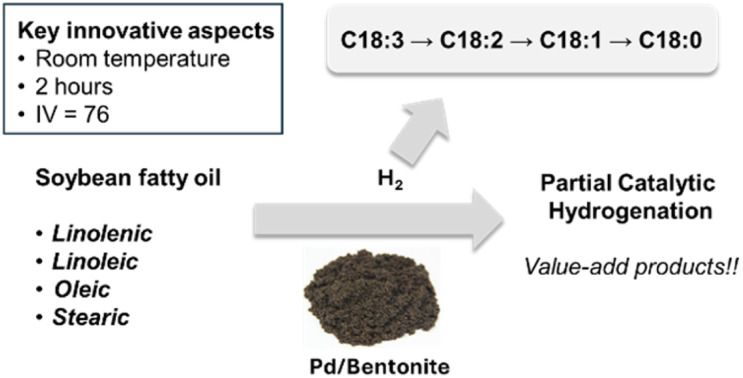

## 1. Introduction

In the food industry, hydrogenation is crucial in saturating the double bonds in vegetable oils.^1,2^ This process transforms the oils from liquid to solid or semi-solid states, enhances their resistance to oxidation, and extends their shelf life.^3^ At an industrial scale, hydrogenation is typically carried out in semi-continuous reactors where the oil is mixed with the metal catalyst (generally 0.05% of a metal–oil ratio). The temperature is then raised between 120–180 °C, and H_2_ is introduced at a pressure of 15 and 50 psi. Once the desired hydrogenation level has been reached, the catalyst is removed by filtration. The oil undergoes a bleaching process to eliminate any trace metal from the final product.^4,5^ During these steps, simultaneous reactions occur,^6^ including saturation of any double bond present, partial hydrogenation, migration of double bonds along the hydrocarbon chain, and the conversion of *cis* to *trans* geometry.^7,8^ These reactions can be favoured by adjusting the conditions under which the hydrogenation occurs, resulting in specific mixtures of fatty oils.^9,10^

From a nutritional perspective, the formation of *trans*-hydrogenated products is considered undesirable, since it is associated with various health issues.^11–13^ Among these health issues are cardiovascular diseases,^14^ obesity,^15^ diabetes,^16^ cancer,^17^ infertility, and complications during fetal development.^18^ However, from an industrial angle, *trans* fats are valuable feedstock for chemical manufacturing^19–25^ and energy production (fuels).^26–31^

In this context, a wide range of catalytic processes have been developed to promote the selective hydrogenation of renewable resources, such as vegetable oils, for the chemical industry. This has enabled the revaluation and diversification of these feedstocks, extending their use beyond the food industry.^22^

Traditional partial hydrogenation, including the industrial process, is performed at high temperatures (150–180 °C)^32^ and pressures between 14.5 and 870 psi.^33–35^ The process commonly employs metal catalysts such as Pd,^36–39^ Pt,^40–42^ Ni,^43–46^ or Cu.^47^ Recently, efforts have shifted towards elaborating supported catalysts containing these noble metals for hydrogenation at temperatures below 120 °C. Examples include Ni–Pd–Ru/graphene,^48^ Ni–Ag/PVP-DB-171/SiO_2_/Fe_3_O_4_,^49^ Ni–Ag/SBA-15,^50^ Pd/graphene,^51^ Pd/Fe_3_O_4_@*n*SiO_2_@*m*SiO_2_,^52^ Pd–Pt/SiO_2_,^53^ Pd–B/γ-Al_2_O_3_,^54^ Pd/diatomite,^55^ Pd/Al_2_O_3_/Al,^56,57^ Pt/diatomite,^58^ Pt/onion-like fullerenes (OLF),^59^ Pt/graphene oxide,^60^ Pt/γ-Al_2_O_3_,^61^ Pt–Ni/SiO_3_,^62^ Pt/ZSM-5,^63^ and Cu–Ag/SBA15.^64^

**Scheme 1:**
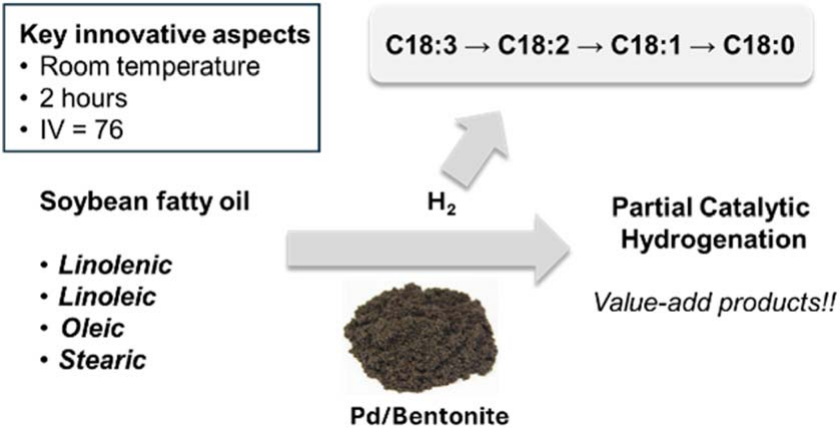
Selective hydrogenation of triacylglycerols.

Building on this background, the objective of this study was to evaluate the catalytic activity of palladium supported on bentonite in the partial hydrogenation of soybean oil at 25 °C and 70 °C. The main goal was to obtain batches of hydrogenated oil that reached iodine values (IV) close to 70 at temperatures below 120 °C (Scheme 1).

This combination of Pd and bentonite has previously been used in various chemical transformations, such as Suzuki coupling,^65,66^ reduction of nitro toluene to obtain aromatic amines,^67^ reductions of azo dyes,^68^ reduction of citral^69^ reduction of benzaldehyde to benzyl alcohol,^70^ acylations of aromatic compounds,^71^ oxidation of benzyl alcohol to benzaldehyde,^72^ and hydrogen storage.^73^ In all of these reactions, the catalytic properties of Pd/bentonite depend on how Pd is supported on the bentonite, which is one of the key factors motivating the present work.

Bentonite consists of clay minerals from the smectite group, with montmorillonite being the primary component. These clays are composed of phyllosilicate layers featuring two main sorts: tetrahedral and octahedral layers.^74^ In particular, the montmorillonite structure consists of two layers of Si^4+^ tetrahedra and one layer of Al^3+^ or Mg^2+^ octahedra, forming a lamina of indefinite extension. The interlaminar space houses hydrated cations, such as Na^+^, K^+^, Ca^2+^, or Mg^2+^ counteracting any charge from isomorphic substitutions. The basal spacing dimension, which includes the thickness of a lamella and the interlaminar distance, can range from 9.6 Å when lamellae are tightly packed to 21 Å when polar molecules are present. Generally, it is approximately 12 Å.^75^

These characteristics contribute to bentonite's high cation exchange capacity and excellent swelling properties, enabling the preparation of a wide variety of catalytically active forms. Recently, cetyltrimethylammonium bromide CTAB was intercalated into the interlayer of bentonite, and Pd(ii) was dispersed in the interior and on the surface to obtain an efficient catalytic system to hydrogenation of polyunsaturated fatty acid methyl ester FAME at 50–90 °C using microwave irradiation and ammonium formate as the hydrogen donor.^76,77^ Despite these initial studies, no heterogeneous process using Pd/bentonite for partial hydrogenation of fatty oil derivatives has been reported.

Here we report the catalytic properties of a Pd/bentonite catalyst, obtained *via* incipient wetness impregnation^78–81^ of Pd(ii) onto bentonite, in the hydrogenation of soybean oil. This different method of preparing Pd/bentonite enabled us to carry out the hydrogenation reaction with H_2_, in contrast to the conditions previously reported for FAMEs.^76,77^

## 2. Experimental

### 2.1. Materials

All solvents and reagents were purchased from Sigma Aldrich and used without further purification. All experiments were performed using deionised water.

### 2.2. Bentonite treatment

The bentonite used in this study was sourced from a mine in Tehuacán, Puebla, Southern Mexico. The clay was treated as follows: 50 g of natural bentonite was grounded in a mortar and suspended in 1 L of deionised water. The suspension was stirred overnight, and the bentonite was separated by centrifugation at 600 rpm for 20 minutes. After repeating this process thrice, the bentonite was dried at 100 °C under vacuum for 72 hours to obtain a white solid.

### 2.3. Catalyst preparation

The catalyst was prepared by impregnating bentonite with a solution of palladium(ii) acetate in ethyl acetate, using the incipient wetness impregnation method.^82^ Two batches of catalysts were prepared with different metal concentrations. The first batch, Bent-Pd-1, was prepared from 2.000 g of bentonite, 0.050 g (0.223 mmol) of Pd(CH_3_COO)_2_ in EtOAc (100 mL), while the second batch, Bent-Pd-2, was prepared from 2.000 g of bentonite, 0.100 g (0.446 mmol) of Pd(CH_3_COO)_2_ in EtOAc (100 mL).

In both cases, 100 mL of EtOAc were added dropwise to the bentonite under constant stirring until a slightly muddy consistency was achieved. This volume was considered the wetting volume of the support. A solution of Pd(CH_3_COO)_2_ in EtOAc (100 mL) was added slowly to a homogeneous mixture. The impregnated bentonites were left to dry at room temperature for 15 hours and were subsequently washed with EtOAc (5 × 50 mL) to recover the unsupported Pd(CH_3_COO)_2_. The washes were monitored by UV-visible spectroscopy until no Pd(CH_3_COO)_2_ was detected in the solution. Palladium acetate has an absorption maximum at 400 nm,^83^ which was used to determine the concentration of Pd recovered after the washings. Once the absorbance was close to zero, the EtOAc organic phases were combined and evaporated under reduced pressure to isolate the amount of recovered Pd(CH_3_COO)_2_. Finally, the catalysts were dried under vacuum at room temperature for 8 hours to remove any residual solvents.

In addition, 0.100 g of the second batch (Bent-Pd-2) were reduced in a reactor at 550 psi of H_2_ pressure to obtain a sample of the catalyst Bent-Pd-3 containing Pd(0). Bent-Pd-3 was used as a control to analyse changes in the natural bentonite after impregnation with Pd(CH_3_COO)_3_.

### 2.4. Characterisation of the catalyst

Powder X-ray diffraction (XRD) was performed using a Siemens D5000 diffractometer with CuKα1 (*λ* = 0.154 nm) at 40 kV and 30 mA. Intensity data were collected from 4° to 70° (2*θ*). Nitrogen adsorption–desorption analysis was conducted at −196 °C using BELSORP-MAX X equipment. The chemical composition of the catalyst was determined by X-ray photoelectron spectroscopy (XPS) with a Thermo Scientific K-Alpha spectrometer. Surface morphology was examined using a JEOL 5900LV scanning electron microscope (SEM), operated at a voltage of 133 eV.

### 2.5. Identification and quantification of acids sites in catalyst

The quantification of acids sites present in the material was determined by FT-IR (Nicolet 750 Spectrometer). The sample was compressed into thin wafers (10 mg cm^−2^) and pre-treated in a quartz cell under vacuum (residual pressure < 2 × 10^−2^ mbar) at 450 °C for 4 hours. The sample was then cooled to room temperature and exposed to pyridine (1 μL) (*P*_eq_ = 2–3 mbar). The excess pyridine was removed under vacuum for 1 hour and evacuated from 50 to 400 °C for 1 hour under vacuum. After each treatment, an FT-IR spectrum was obtained.^84^

### 2.6. General procedure for hydrogenation of soybean oil

Catalytic hydrogenation of soybean oil was carried out in a Parr batch reactor Serie 4760 (160 mL). The reactor was loaded with 0.100 g of Pd catalyst powder and 10 g of soybean oil. After performing three N_2_ purges, the reaction mixture was heated to the required temperature. The N_2_ was then replaced with H_2_ under constant stirring.

### 2.7. Analysis of fatty acid content by GC

The fatty acid content in the starting oils and the reaction products was determined using gas chromatography (GC). FAMEs (C:12–C:24) standards were used for the composition and evaluation of the produced oils after derivatization.

FAMEs were prepared by saponification of a fat aliquot with a 0.5 N KOH solution in MeOH, followed by esterification with MeOH (2 mL) in the presence of BF_3_·OEt_2_ (2 mL). For the saponification reaction, 120 mg of hydrogenated oil was weighed, and 2 mL of KOH solution was added. This mixture was heated in an oil bath at 70 °C until a single phase was achieved. After cooling, HCl (5 mL, 1 N) was added dropwise until an acidic pH of 5 was reached. The organic phase was extracted using hexane (3 × 2 mL) and dried with anhydrous Na_2_SO_4_.

GC samples were analysed using an Agilent HP 6890 Series GC system with an FID detector and an Agilent SP-2560 column (75 m × 0.18 mm × 0.14 μm). The temperature ramp profile was an initial hold at 140 °C for 5 minutes, ramped up to 240 °C at a rate of 4 °C min^−1^, and held at 240 °C for 2 min, with a gas flow rate of 1.6 mL min^−1^ (constant flow) using H_2_ as the carrier gas. The injector temperature was 250 °C, and the FID was set at 250 °C.

### 2.8. Analysis of fatty acid content by NMR

A ^1^H NMR analysis of the initial soybean oil was established as a standard, and any hydrogenation experiment was compared to it. ^1^H NMR spectra were obtained using a Bruker Avance spectrometer (400 MHz). Each sample (20 mg) was dissolved in CDCl_3_ with a small amount of TMS as an internal standard. The integral values were used to calculate the number of olefinic protons as equivalence of the number of double bonds in the sample.

### 2.9. Regeneration and reuse of catalyst

After the hydrogenation reaction, the used catalyst was removed by centrifugation and washed successively with methanol (3 × 15 mL), ethyl acetate (3 × 15 mL) and hexane (3 × 15 mL) to remove any residual product. The catalyst was then dried at room temperature under vacuum for 18 hours and subsequently characterised by XRD to confirm its homogeneity before reuse. To carry out a reaction with the recovered catalyst, the general hydrogenation procedure protocol described above was followed.

## 3. Results and discussion

### 3.1. Pd content in the catalyst

**Fig. 1:**
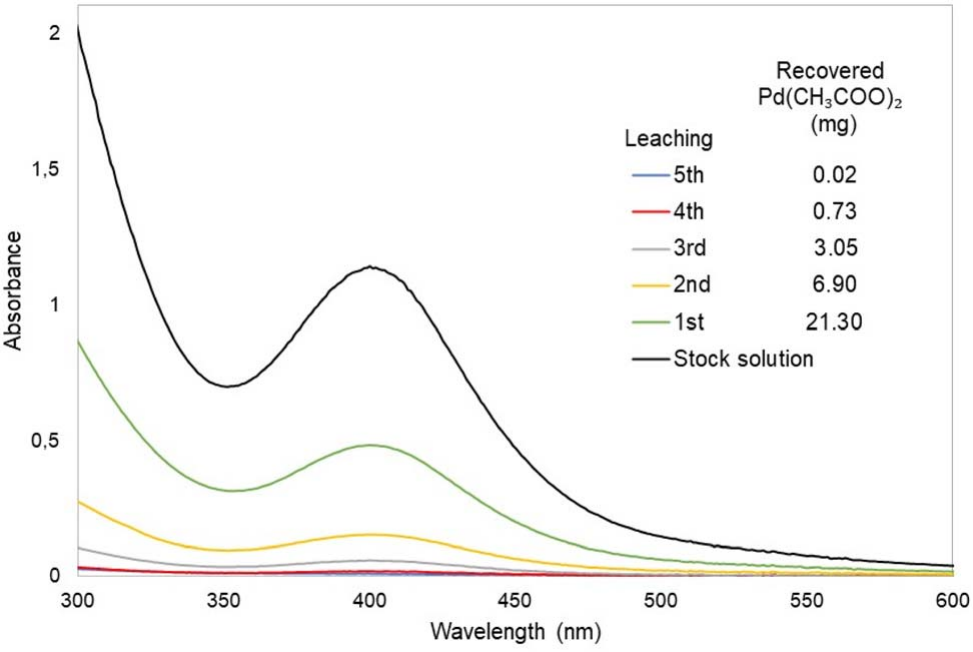
UV-vis spectra of Pd(CH_3_COO)_2_ solution after washing of Bent-Pd-2.

As part of the catalyst preparation process, bentonite was washed with ethyl acetate to remove any palladium that was not supported on the catalyst. The amount of palladium recovered from the washings was then quantified using UV-vis spectroscopy and gravimetry. The difference between the palladium acetate initially used in the impregnation process and the recovered palladium acetate allowed for the determination of the amount of metal incorporated into the bentonite. For example, 1.00 g of Bent-Pd-1 contains 0.004 g of Pd (0.4%), while 1.00 g of Bent-Pd-2 contains 0.016 g of Pd (1.6%). Fig. 1 shows the absorption spectral changes in the region from 280 to 550 nm, associated with the gradual decrease in palladium acetate concentration after each washing of Bent-Pd-2 with ethyl acetate. The absorbance of the fourth and fifth washings is close to zero, suggesting that the palladium leaching process has concluded.

### 3.2. Characterization of the catalyst

#### 3.2.1 X-ray diffraction (XRD)

**Fig. 2:**
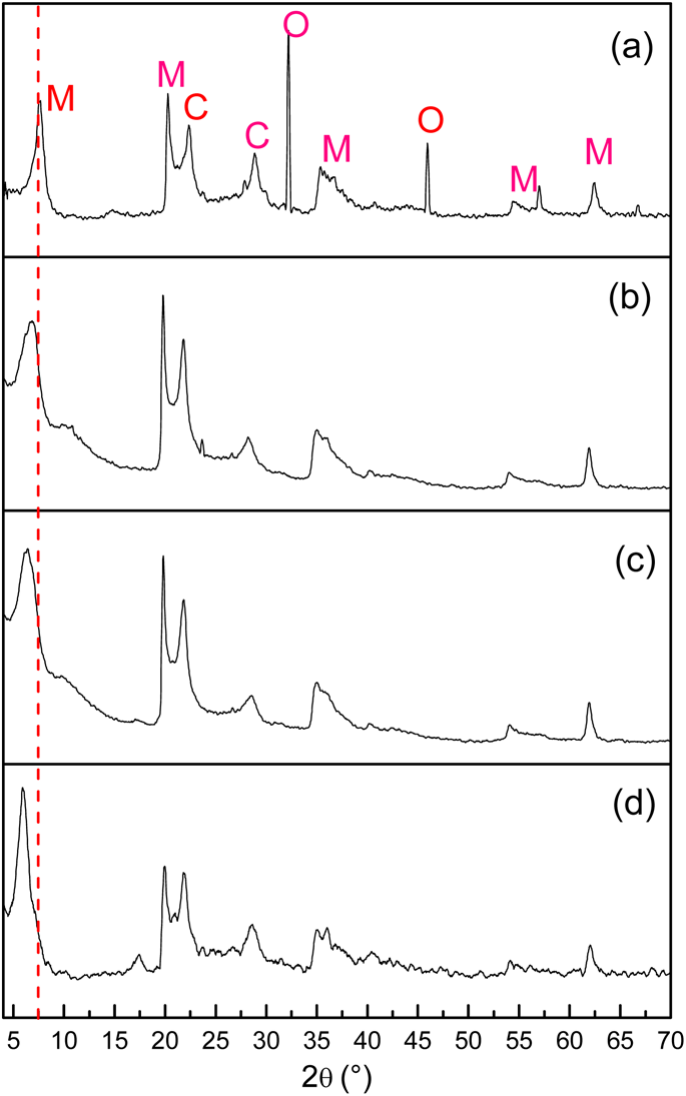
XRD of (a) Nat-Bent, (b) Bent-Pd-1, (c) Bent-Pd-2 and (d) Bent-Pd-3. M: montmorillonite, C: cristobalite, and O: iron oxide.

Fig. 2 displays the diffractograms obtained from bentonite at three stages of preparation: (a) unmodified (Nat-Bent), (b, c) impregnated with Pd(CH_3_COO)_2_ (Bent-Pd-1 & Bent-Pd-2), and (d) after reduction of the introduced Pd (Bent-Pd-3). The diffraction pattern of the bentonite (Fig. 2a) indicates its mineral composition is predominantly montmorillonite, with cristobalite and iron oxide as additional crystalline phases. It can be inferred that iron oxide is likely an impurity rather than an intrinsic component of the bentonite, as its signal disappears upon impregnation and subsequent washing.

The most intense peak (montmorillonite) was utilised to calculate crystallite size and basal spacing. Changes in this peak provide fundamental information about modifications in the bentonite structure. A dotted line was added at the central value of this signal (2*θ* = 7.2°) as a reference for comparison in the other spectra. The natural bentonite has an average crystallite size of 10.6 nm and a basal spacing of 12.24 Å, characteristic of smectite minerals with an adsorbed molecular layer of water between their sheets.

In the Pd-supported samples' spectra, no characteristic diffraction peaks of the metal (Pd) were observed (2*θ* = 40.4°). However, broadening and displacement of the main peak of montmorillonite to smaller angles were observed. The absence of signals for Pd suggests that its particles are present at very low concentrations and are uniformly distributed in the bentonite, complicating their detection.

Any change in the montmorillonite peak region indicates modifications in the crystal structure of bentonite (Fig. 2b and c). The broadening of the peaks suggests that the distance between layers is variable and illustrates a partially delaminated structure. This may result from the amount of Pd present, leading to less effective pillaring and a less ordered structure with greater variation in the spacing between layers. Notably, the reduced sample Bent-Pd-3 exhibits the least broadening, a result consistent with the presence of small Pd(0) particles well dispersed in the bentonite. Thus, the elimination of acetate from the precursor salt facilitates the interaction of palladium with the clay molecules, reconstituting its ordered structure.

The shift of the most intense peak corresponding to montmorillonite (2*θ* = 7.2°) towards smaller angles in the modified bentonite samples (Fig. 1a–d) indicates an increase in basal spacing and intercalation of Pd in the interlaminar area of the bentonites. As shown in Table 1, a decrease in crystallite size is observed when impregnated with Pd(CH_3_COO)_2_ due to the intercalation of Pd in the interlaminar space. A partially delaminated structure with variable crystallite sizes throughout the material is confirmed.

**Table 1:** Comparison of crystallite size *versus* basal spacing

Entry	Catalyst	Basal spacing (Å)	Crystallite size (nm)
1	Nat-Bent	12.24	10.6
2	Bent-Pd-1	12.53	7.8
3	Bent-Pd-2	14.56	7.0
4	Bent-Pd-3	14.76	7.7

#### 3.2.2 Textural properties of bentonites

**Fig. 3:**
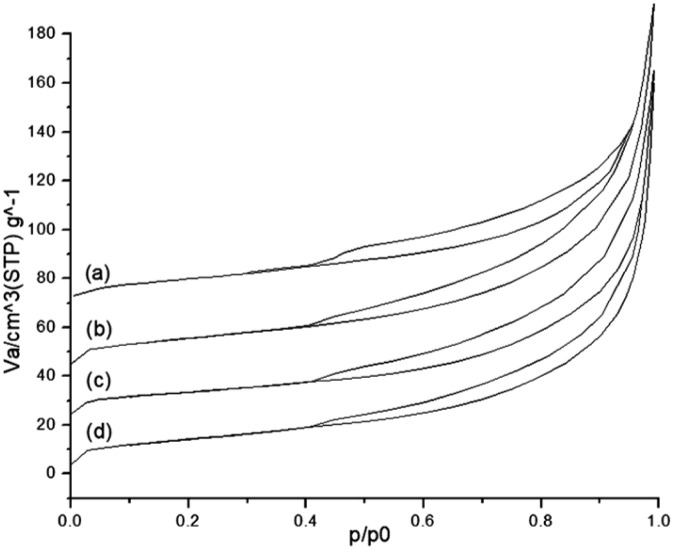
Nitrogen adsorption–desorption isotherm of (a) Nat-Bent, (b) Bent-Pd-1, (c) Bent-Pd-2 and (d) Bent-Pd-3.

N_2_ adsorption–desorption is illustrated in Fig. 3. The isotherms of natural bentonite (Nat-Ben) and modified bentonite after Pd impregnation are type IV (mesoporous solids). The hysteresis loop corresponds to type H3 associated with plate-shaped aggregates exhibiting slit-shaped pores—a characteristic of bentonites. Using these adsorption–desorption data and applying the Brunauer–Emmett–Teller BET method, the textural characterisation of all samples was carried out (Table 2).

**Table 2:** Textural properties of bentonites

Entry	Catalyst	BET area (m^2^ g^−1^)	Pore volume (cm^3^ g^−1^)	Pore size (nm)
1	Nat-Bent	48.023	0.2420	20.160
2	Bent-Pd-1	52.374	0.2299	17.555
3	Bent-Pd-2	56.159	0.2252	18.650
4	Bent-Pd-3	51.539	0.2316	18.750

For natural bentonite (Nat-Bent), a surface area of 48.02 m^2^ g^−1^ was obtained, which is relatively higher than that described for this type of material.^74,75^ The total pore volume was 0.2420 cm^3^ g^−1^, with a pore diameter of 20.16 nm, corresponding to a mesoporous solid according to IUPAC classification. For the modified bentonites samples, the BET surface area increased due to the intercalation of Pd(CH_3_COO)_2_ between the clay lamellae, acting as pillars that maintain separation between the lamellae and thereby expose the interior surface of the bentonite to N_2_. Both the total pore volume and the average diameter decreased from natural bentonite to modified bentonite samples (Table 2), suggesting the deposition of Pd particles occurred within the pores.

#### 3.2.3 X-ray photoelectron spectroscopy (XPS) of bentonites

**Fig. 4:**
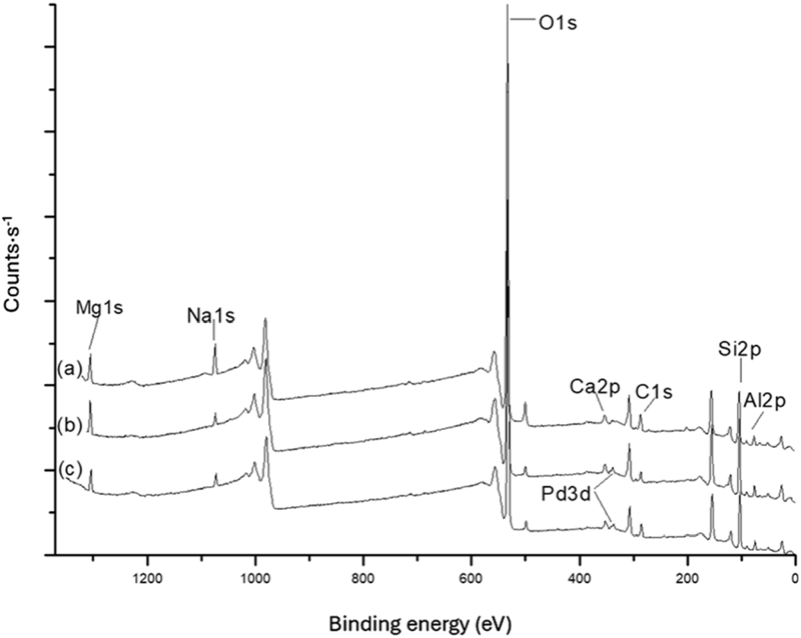
XPS of (a) Nat-Bent, (b) Bent-Pd-2 and (c) Bent-Pd-3.

Fig. 4 presents the XPS spectrum of natural bentonite before and after impregnation with Pd (Bent-Pd-2), and reduction (Bent-Pd-3). In all samples, silicon, oxygen, and aluminium—elements typical of aluminosilicates—were observed. The addition of Pd(CH_3_COO)_2_ did not result in significant changes to the binding energies of the primary bentonite elements. The only notable change was the appearance of a peak corresponding to Pd in the spectra of Bent-Pd-2 and Bent-Pd-3 confirming the successful impregnation of Pd within the bentonite matrix.

**Fig. 5:**
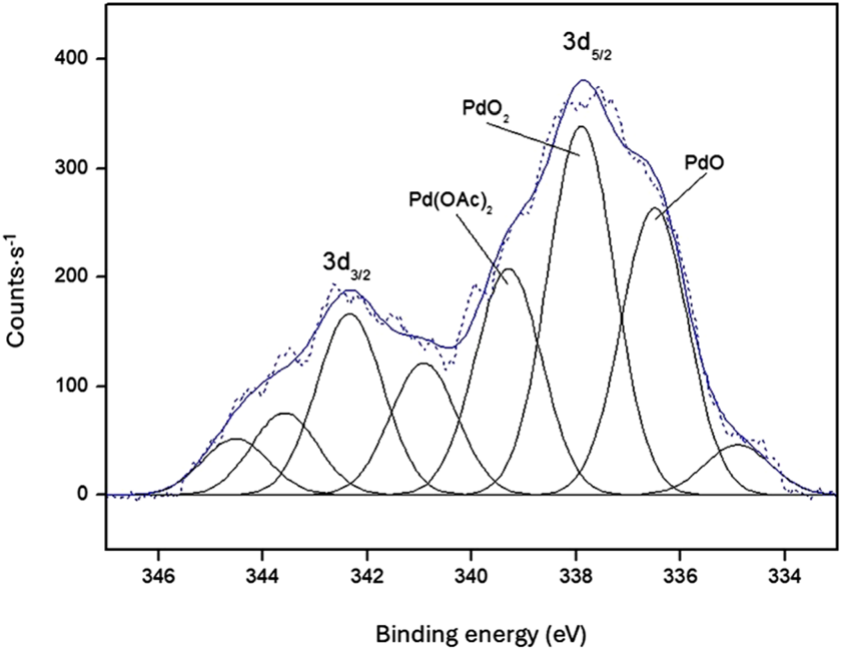
High-resolution XPS spectra of Bent-Pd-2.

The high-resolution Pd 3d spectrum of Bent-Pd-2 is shown in Fig. 5. Deconvolution of the 3d_5/2_ peak reveals the presence of three distinct forms of Pd on the surface of bentonite. The main peaks, centred at 337.9 eV and 336.5 eV, are characteristic of PdO, while the shift at 339.3 eV is associated with Pd(CH_3_COO)_2_. The small peak at 334.9 eV is a satellite peak of oxidised Pd species.

The chemical composition of natural bentonite and modified bentonites as determined by XPS is listed in Table 3. The most notable change is the presence of Pd in the modified bentonites, along with a decrease in Na content, meaning that the introduced Pd displaced the sodium cations.

**Table 3:** Atomic composition of bentonites determined by XPS

Entry	Elem.	Atomic%			
		Nat-Bent	Bent-Pd-1	Bent-Pd-2	Bent-Pd-3
1	O 1s	55.39	58.83	58.39	56.77
2	Si 2p	23.98	24.54	25.83	25.36
3	Al 2p	5.93	6.10	6.44	6.05
4	C 1s	7.94	4.37	3.62	6.38
5	Ca 2p	0.98	1.12	0.94	1.17
6	Mg 1s	2.95	3.72	3.26	2.65
7	Na 1s	2.83	1.22	1.31	1.38
8	Pd 3d	0.00	0.10	0.20	0.24

#### 3.2.4 Acidic properties

**Fig. 6:**
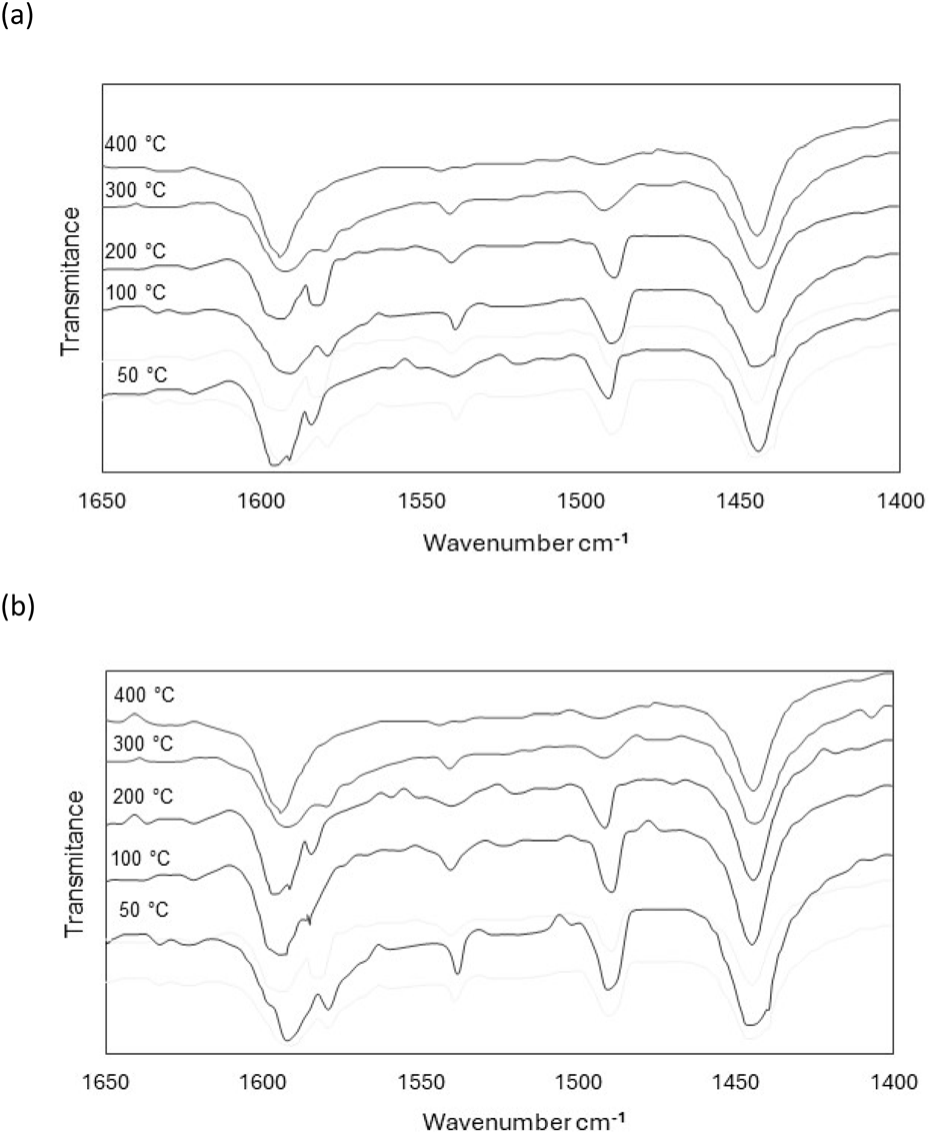
FT-IR spectra of (a) Nat-Bent and (b) Bent-Pd-2 after pyridine desorption at different temperatures: 50 °C, 100 °C, 200 °C, 300 °C, and 400 °C.

Adsorption–desorption of pyridine at different temperatures, monitored by FT-IR spectroscopy, was used to evaluate the nature and the concentration of acid sites (Brøensted and Lewis) of Nat-Bent and Bent-Pd-2. Fig. 6 shows the FT-IR spectra for both samples, where the characteristic bands of the pyridinium ion at 1595, 1540 and 1490 cm^−1^ (Brønsted acid sites), and pyridine coordinated to Lewis acid sites at 1580, 1490 and 1445 cm^−1^, were observed.^84^ The concentration of both types of acid sites was estimated from the intensities of the bands at 1490 and 1445 cm^−1^ using eqn (1):

1 *n*_*i*_ = *A*_i_*a*_c_/*ε*_i_*m*

where: *n*_*i*_ is the amount of type *i* acid sites (μm mol g^−1^). *A*_i_ is the integrated absorbance in cm^−1^. *a*_c_ is the cross-sectional area in square centimetres of the wafer. *ε*_i_ is the integrated molar extinction coefficient in cm μm mol^−1^. *m* is the mass of the sample.

As shown in Table 4, there was no significant difference in the acidity of the samples with and without Pd. This suggests that the low concentration of Pd in the bentonite does not influence the final acidity of the material.

**Table 4:** Concentration of acid sites as calculated after pyridine desorption at different temperatures

Sample	Temp. (°C)	Brønsted (μmol g^−1^)	Lewis (μmol g^−1^)	Total	B/L ratio
Nat-Bent	50	2.72	5.31	8.03	0.51
	100	1.89	3.95	5.84	0.47
	200	0.92	1.87	2.79	0.49
	300	0.49	0.89	1.38	0.55
	400	0.26	0.55	0.81	0.47
Bent-Pd-2	50	2.75	5.34	8.09	0.51
	100	1.91	3.98	5.89	0.48
	200	0.97	1.91	2.88	o.47
	300	0.52	0.95	1.47	0.54
	400	0.23	0.49	0.72	0.46

#### 3.2.5 Scanning electron microscopy (SEM)

**Fig. 7:**
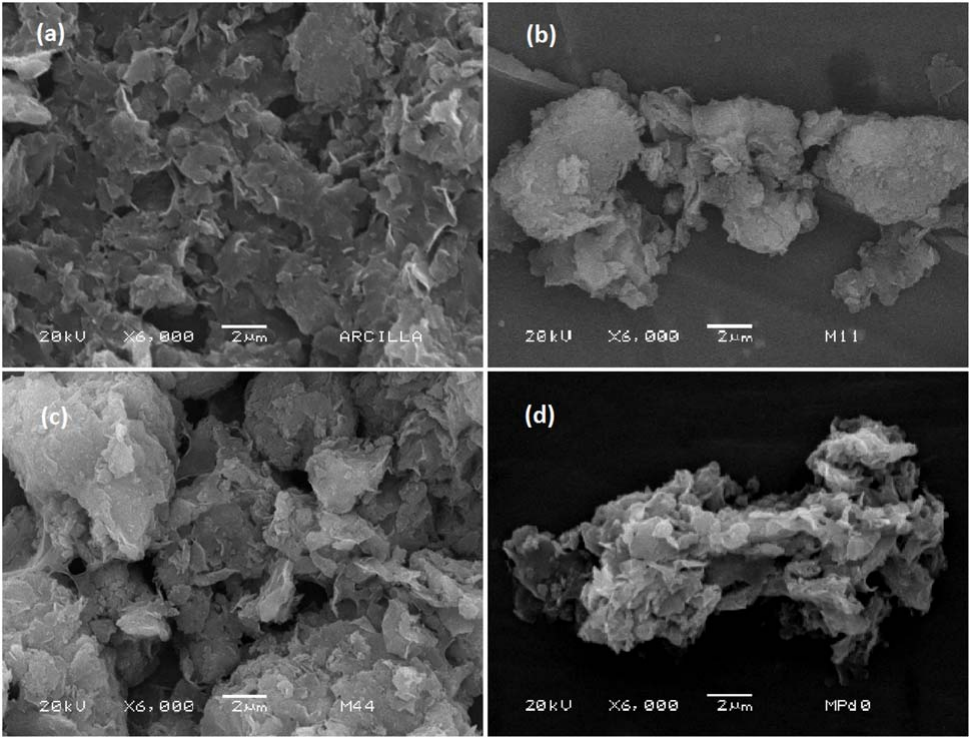
SEM images of (a) Nat-Bent, (b) Bent-Pd-1, (c) Bent-Pd-2 and (d) Bent-Pd-3.

The morphology of natural and modified bentonites is depicted in Fig. 7. The micrograph corresponding to natural bentonite (Fig. 7a) displays irregular particles forming small aggregates with a relatively smooth surface. The impregnation of Pd resulted in minimal alteration in the morphology of the bentonite, leading to the formation of larger aggregates (Fig. 7b–d).

### 3.3. Characterisation of soybean oil

#### 3.3.1 Analysis of fatty acids

**Fig. 8:**
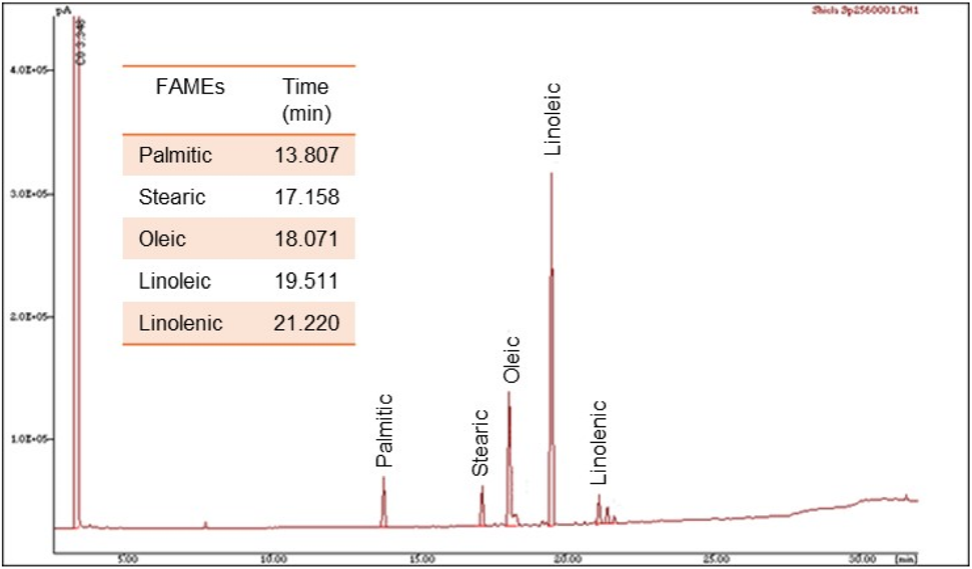
GC profile of the raw FAMEs.

The initial soybean oil was analysed using gas chromatography (GC) to establish a standard. As shown in Fig. 8 and Table 5, the GC method determined the composition of the different FAMEs.

**Table 5:** Fatty acid composition of the initial soybean oil, obtained by CG

	Composition and content					
FAMEs	16:0	18:0	18:1 (*cis*)	18:2 (*cis*)	18:2 (*trans*)	18:3 (*cis*)
%	14.64	4.09	22.92	51.22	0.59	6.54

In addition, the IV index measures the unsaturation of fatty acids. It represents the mass (g) of iodine consumed per 100 g of oil. It can be calculated from the fatty acid composition according to the AOCS norm Cd 1c-85.^85–88^ For our purpose, the determination of IV was based on the concentration FAMEs by GC, as either molar or mass fractions. Thus, the resulting IV was calculated using the following equation (eqn (2)):

2 IV = (*x*_C18:1_ × 0.860) + (*x*_C18:2_ × 1.732) + (*x*_C18:3_ × 2.616)

where *x*_C*y*:*z*_ is the percentages of the different unsaturated compounds found in vegetable oils, multiplied by the factors for each FAME component. Consequently, the IV determined for the soybean oil was 127.

**Fig. 9:**
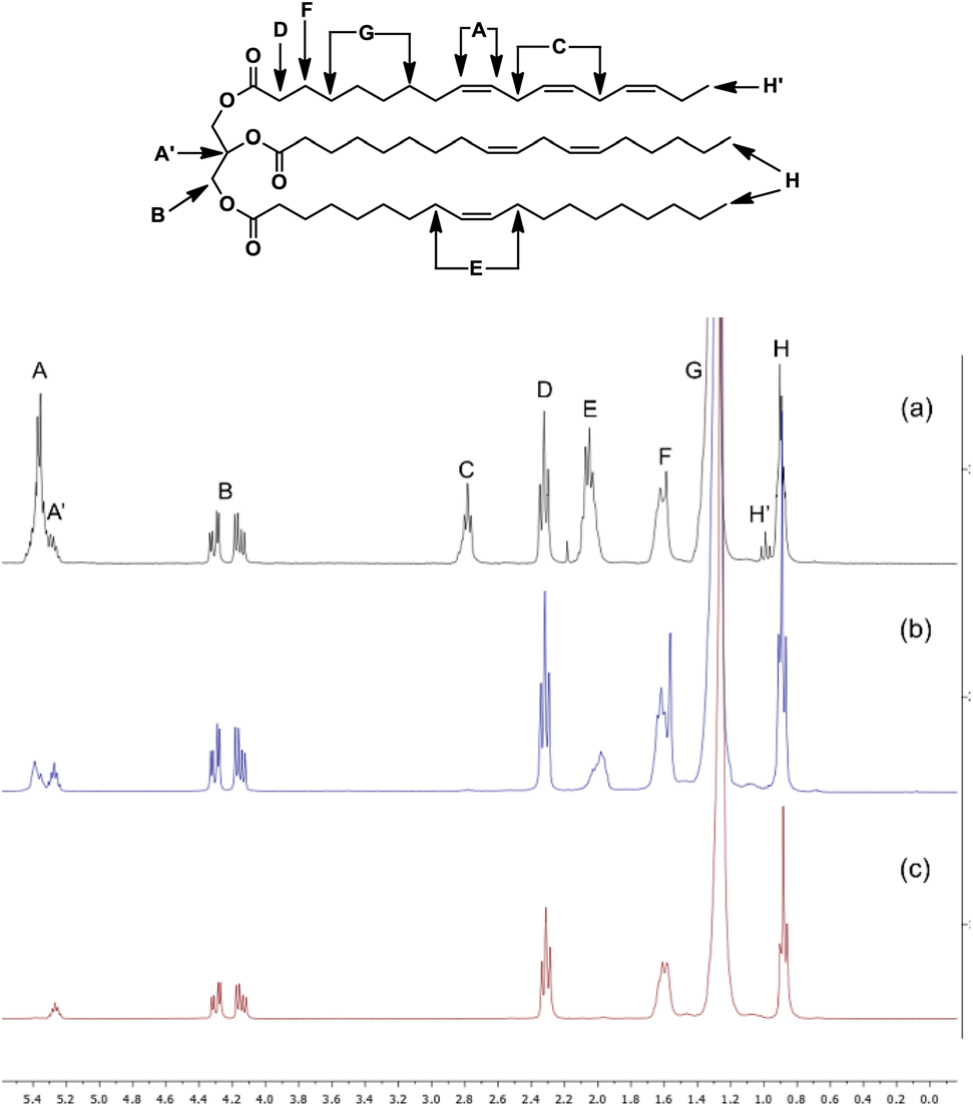
^1^H NMR of (a) soybean oil, (b) hydrogenated soybean oil after 60 min at 70 °C, and (c) hydrogenated soybean oil after 120 min at 70 °C.

In addition to GC, the degree of unsaturation was also assessed using ^1^H NMR. Fig. 9 shows the spectra of soybean oil, where eight distinct types of protons are identified, labelled A–H and outlined in Table 6. The main changes in the spectra of the FAMEs, compared to those of the initial soybean oil, were observed in signals A, C, E, and H′, which correspond to protons located near double bonds.

**Table 6:** Signal assignment for proton types in the ^1^H NMR spectrum of soybean oil

Signal	Proton signals
A	Olefinic protons
A′	The central proton of glycerol
B	Four methylene protons of glycerol
C	Diallylic protons
D	Six α-carbonyl protons
E	Allylic protons
F	Six β-carbonyl protons
G	Methylene protons
H	Methyl protons
H′	ω-3 fatty acid methyl protons

The area of each signal in the spectrum is proportional to the number of equivalent protons contributing to the peak. By integrating these signals, we were able to calculate the area per proton (eqn (2)), the average molecular weight (eqn (3)), and the iodine value (IV, eqn (4)). The IV is directly related to the proportion of olefinic hydrogens present in the sample. In our study, the IV calculated for the initial soybean oil was 129 (eqn (3)–(5)).3
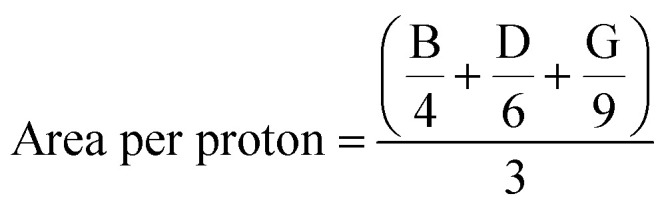
4
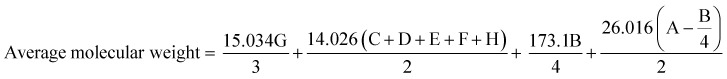
5
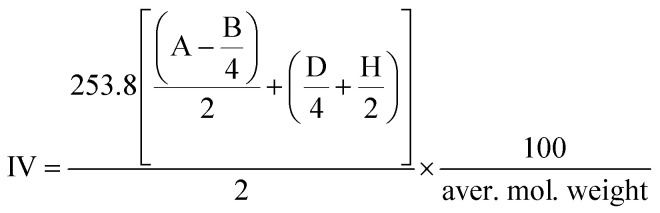


**Fig. 10:**
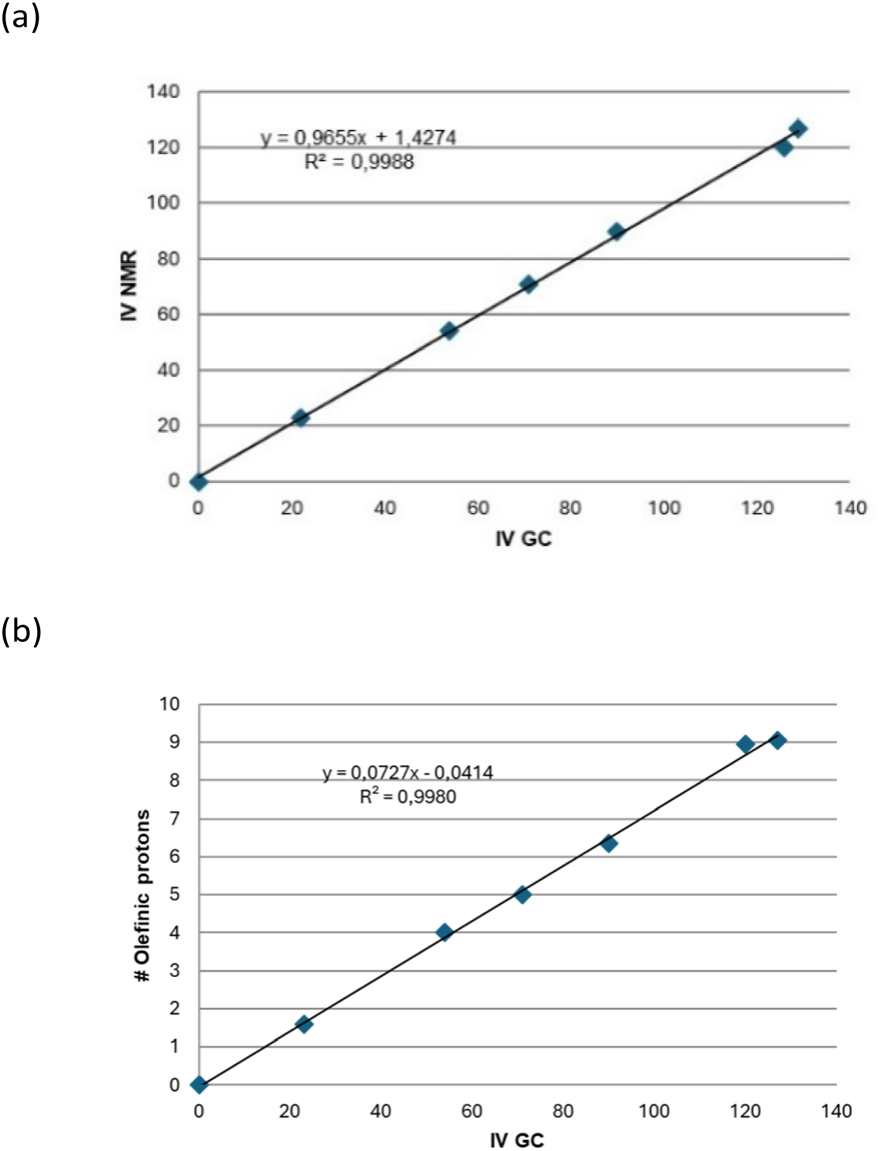
(a) Correlation between IV obtained by GC and ^1^H NMR and (b) correlation between the number of olefinic protons and the IV of CG.

To corroborate the feasibility of ^1^H NMR to track the hydrogenation reaction, the IV obtained *via* GC of FAMEs was compared graphically with those derived from the ^1^H NMR spectra. As previously described,^89,90^ a linear relationship is observed between ^1^H NMR data and GC data (Fig. 10). Therefore, either of these methods can be used interchangeably to determine the degree of unsaturation present in the samples.

### 3.4. Catalytic activity of modified bentonites

#### 3.4.1 Effect of the pressure and metal loading

To establish the optimal reaction conditions, we initially focused on studying the effects of the pressure and metal loading on the complete hydrogenation of vegetal oil. At this stage of the study, the efficiency of the two prepared catalysts (Bent-Pd-1 and Bent-Pd-2) was evaluated. In all experiments, the amount of catalyst used was kept constant (0.100 g). However, the Pd concentration varied between the catalysts, Bent-Pd-1 contains 0.4% Pd and Bent-Pd-2 contains 1.6% Pd. The temperature (70 °C) was selected to ensure that soybean oil remained in a liquid state, while the pressure at which the hydrogenation was conducted was varied. The efficiency of the catalysts was evaluated based on the achievement of 100% stearic acid, with the result being the variation in reaction time required to reach this 100%. The results of these experiments are shown in Table 7.

**Table 7:** Reaction conditions investigated to hydrogenation of soybean oil^a^

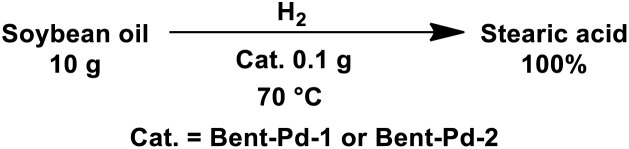			
Entry	Pressure (psi)	Time (min)	
		Bent-Pd-1 (0.004% based on Pd content)	Bent-Pd-2 (0.016% based on Pd content)
1	150	2520	720
2	250	2160	480
3	350	1440	390
4	450	720	270
5	550	480	120
6	750	460	120
7	1050	430	120

a Conversion calculated by GC.

**Fig. 11:**
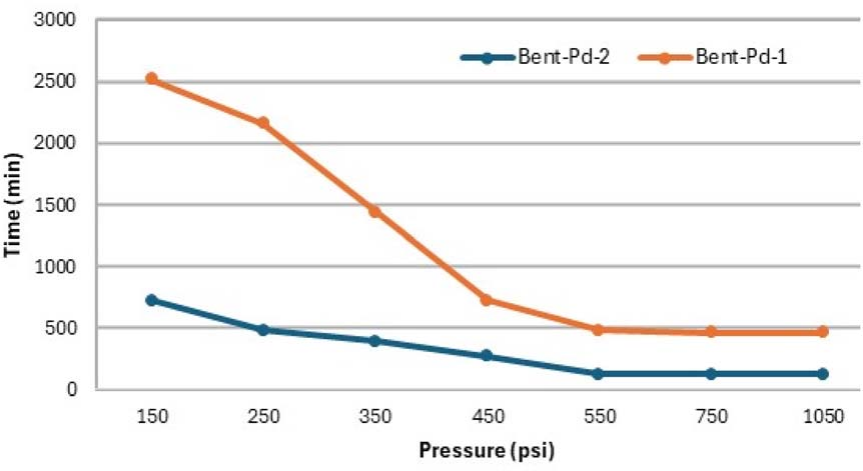
Evolution of the hydrogenation reaction as the H_2_ pressure is varied.

Thus, with Bent-Pd-1, complete hydrogenation of the vegetable oil was achieved in a longer time compared to the use of Bent-Pd-2 (Table 7, entries 1–7). It is evident that, in both cases, the reaction time decreases as the pressure increases. The shortest reaction time, 120 minutes, was observed at 550 psi of H_2_ using the Bent-Pd-2 catalyst (Table 7, entry 5). Fig. 11 shows the trend in reaction time as the hydrogenation pressure is varied.

#### 3.4.2 Partial hydrogenation of soybean oil

**Fig. 12:**
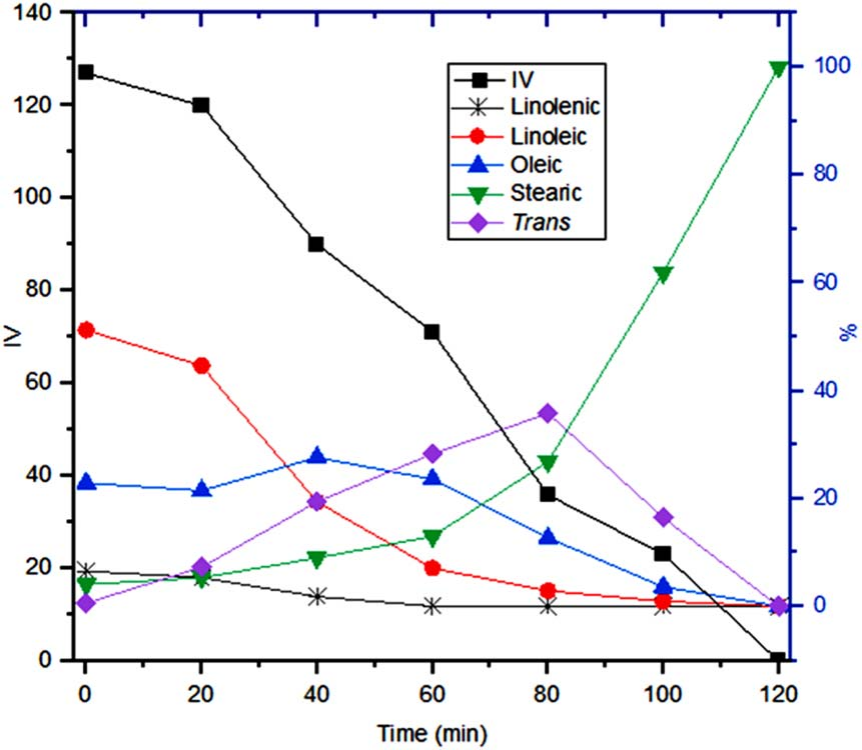
Profile of soybean oil during hydrogenation using Bent-Pd-2 catalyst (0.100 g) and determined by GC. Reaction conditions: *T* = 70 °C, *P* = 550 psi, %Pd = 0.016, 10 g oil.

Given that the aim of this study is the partial hydrogenation of vegetable oil to achieve IV close to 70, the next step was to analyse the progress of the reaction at 550 psi of H_2_, using the Bent-Pd-2 catalyst at 70 °C. The reaction was monitored by GC every 20 minutes until 100% conversion of the vegetable oil to stearic acid was observed. Fig. 12 depicts the evolution of the oil composition during hydrogenation. At first (20 min), minimal changes in the soybean oil composition were observed due to the reduction and pre-hydrogenation of the Pd in the catalyst during this initial period.

Linolenic acid was completely hydrogenated or isomerised within the first 60 minutes of the reaction. Meanwhile, linoleic acid gradually decreased (Fig. 12, grey and red lines). Both oleic acid and *trans* fatty acids (TFA) initially reached maximum concentrations of 28% at 40 minutes and 36% at 80 minutes; respectively. After these maxima, both compounds steadily decreased until complete hydrogenation was achieved. This increase is attributed to the partial hydrogenation of linoleic and linolenic acids, as they produce oleic acid and TFAs as by-products. In the case of TFA formation, this process also involves the isomerisation of oleic acid. TFAs were analysed as a whole, disregarding the position of the double bond in the hydrocarbon chain (Fig. 12, blue and purple lines).

Stearic acid exhibited a steady rise during the first 80 minutes of the reaction, followed by a surge concurring TFA concentration reduction. Thus, hydrogenation of TFA contributed significantly to stearic acid escalation (Fig. 12, green line). Calculated IV values by GC and ^1^H NMR had a minimal variation at the beginning until a continuous decline reached 0.0 after 2 hours (Fig. 12, black line). After 60 minutes of reaction, an IV of 71 (GC) or 72 (NMR) was reached (black line).

**Fig. 13:**
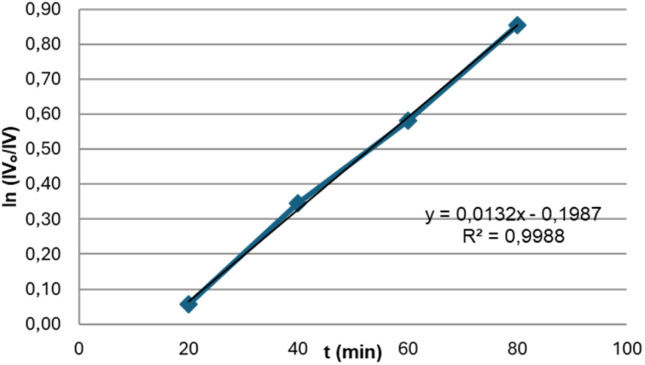
Decrease in IV with respect to time.

Since the saturation of double bonds follows first-order kinetics concerning the decrease in IV value,^91–93^ as illustrated in Fig. 13, this reduction was used to determine the activity of the catalyst during hydrogenation, following eqn (6):6
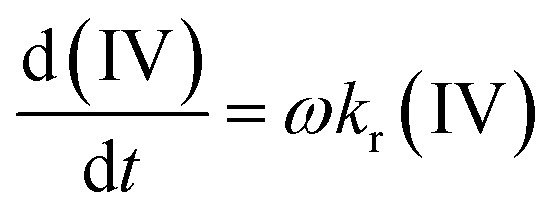
where: *k*_r_ is the reaction constant under specific conditions, *ω* is the ratio (g metal per kg fatty oil).

For the catalyst used in the hydrogenation tests at 70 °C, *k*_r_ was 0.08. This indicates that with 1 g of Pd (corresponding to 62.5 g of catalyst), it is possible to hydrogenate 80 g of fatty oil per minute.

#### 3.4.3 Effect of temperature on catalytic activity

After assessing the behaviour of the reaction at 70 °C, tests were conducted at 25 °C to evaluate the effect of reduced temperature on the hydrogenation products using the Bent-Pd-2 catalyst.^94^ The tests were conducted for 60 to 240 minutes, after which the reactions were stopped. Table 8 summarises the results of the different tests conducted, while activity measured based on the decrease in IV values, expressed as:7
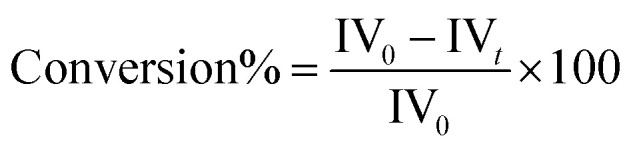
where: IV_0_ is the iodine value before hydrogenation reaction, IV_*t*_ is the iodine value after hydrogenation reaction.

**Table 8:** Hydrogenation reaction at different temperatures and time using Bent-Pd-2 catalyst

Fatty acid%	Original oil					
Stearic	4.09	5.35	16.57	100	13.01	100
Oleic	22.92	22.13	30.49	0	23.61	0
Linoleic	51.22	38.23	13.91	0	7.09	0
Linolenic	6.54	0.98	00	0	0.00	0
*Trans*	0.59	5.31	11.25	0	28.33	0

IV by GC	127	119.00	**76**	0	**71**	0
IV by NMR	129	120	**78**		**72**	0
Conversion (%)		6.2	39.70	100	44.0	100
SR		2.22	5.98		9.66	0.00
Temperature (°C)		25	25	25	70	70
Time (minutes)		60	120	240	60	120

**Fig. 14:**
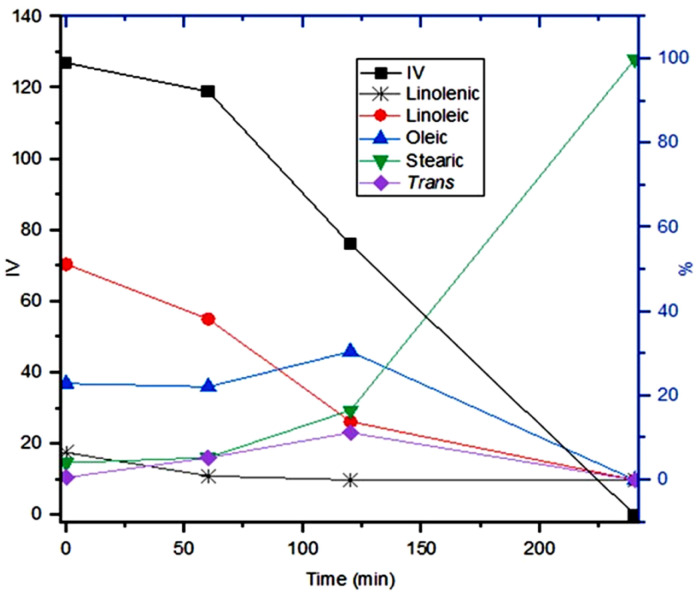
Profile of soybean oil during hydrogenation using Bent-Pd-2 catalyst (0.100 g) and determined by GC. Reaction conditions: *T* = 25 °C, *P* = 550 psi, %Pd = 0.016, 10 g oil.

Fig. 14 depicts the evolution of the oil composition during hydrogenation. Linolenic acid was completely hydrogenated or isomerised after 120 minutes of the reaction. Linoleic acid exhibited a gradual diminution until it was fully hydrogenated after 240 minutes (Fig. 14, grey and red lines). Oleic acid and TFA initially increased, reaching maximum concentrations of 30.49% and 11.25%, respectively, at 120 minutes. After these peaks, both compounds steadily decreased until complete hydrogenation was achieved (Fig. 14, blue and purple lines). Stearic acid exhibited a steady increase during the first 120 minutes of the reaction, followed by an accelerated rise until complete hydrogenation was achieved (Fig. 14, green line). After 120 minutes of reaction, an IV of 76 (GC) or 78 (NMR) was reached (black line).

#### 3.4.4 Comprehensive comparison of results

A decrease in temperature to 25 °C reduced the formation of TFA and the catalytic activity. The TFA content decreased from 28.33% at 70 °C to 11.25% at 25 °C, representing a reduction above 50%. The reduction in catalytic activity was particularly evident in the reaction time, which doubled to achieve a similar IV value at 25 °C (120 minutes). Notably, this change in the temperature did not lead to a substantial increase in stearic acid content; instead, it resulted in a higher production of oleic acid. Furthermore, as shown in Table 8, the reaction at 25 °C for 1 hour resulted in the lowest conversion in this study.

#### 3.4.5 The selectivity ratio (SR)

Table 8 presents the selectivity ratio SR for both temperatures at which the reaction was studied. In this case, SR expressed the preferential hydrogenation of linoleic acid to produce oleic acid and/or TFA. The selectivity of the catalyst was calculated using eqn (8):^35^8
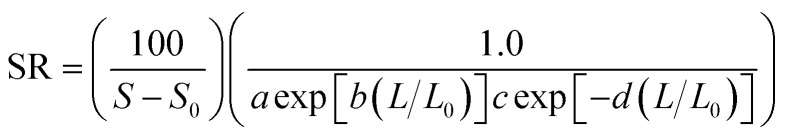
where: *S*_0_ = concentration of stearic acid at the beginning of the process, *S* = concentration of stearic acid at the end of the process, *L*_0_ = concentration of linoleic acid at the beginning of the process, *L* = concentration of linoleic acid at the end of the process.

For soybean oil the values of the constants are: *a* = 1.260, *b* = 2.065, *c* = 0.771 and *d* = 2.299.

Analysis of oil composition after 60 minutes (70 °C, with an IV 71) and 120 minutes (25 °C, with an IV 76) indicates that the catalyst's selectivity is higher at 70 °C. This result suggests that the hydrogenation of linoleic acid occurs more rapidly at this temperature, and other unsaturated products, including positional and geometric isomers, associated with the increased production of TFAs, were produced (Table 8).^95^

**Scheme 2:**
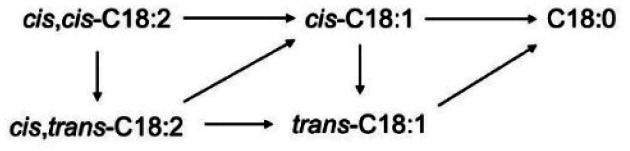
Reaction pathway for the hydrogenation of soybean oil.

Thus, under our reaction conditions, the overall hydrogenation process involves the saturation of *cis*,*cis*-C18:2 to C18:0, passing through the formation of *cis*-C18:1. The process also includes the isomerisation of *cis*,*cis*-C18:2 to *cis*,*trans*-C18:2 and *cis*-C18:1 to *trans*-C18:1. Finally, the hydrogenation process involves the hydrogenation of *cis*,*trans*-C18:2 to *cis*-C18:1 and *trans*-C18:1 to C18:0 (Scheme 2).^96,97^

#### 3.4.6 Effect of the support

To investigate the impact of supporting Pd on bentonite, hydrogenation was performed using Pd(AcO)_2_ powder. This test demonstrated a significant decrease in IV values compared to the test with a supported catalyst. When the reaction was carried out in the presence of Pd(AcO)_2_ at 70 °C and 550 psi for 2 h, the IV value reached 116. This finding confirms that the increased activity achieved by depositing the active metal on support enhances exposure the reaction medium.

#### 3.4.7 Reuse of the catalyst

**Fig. 15:**
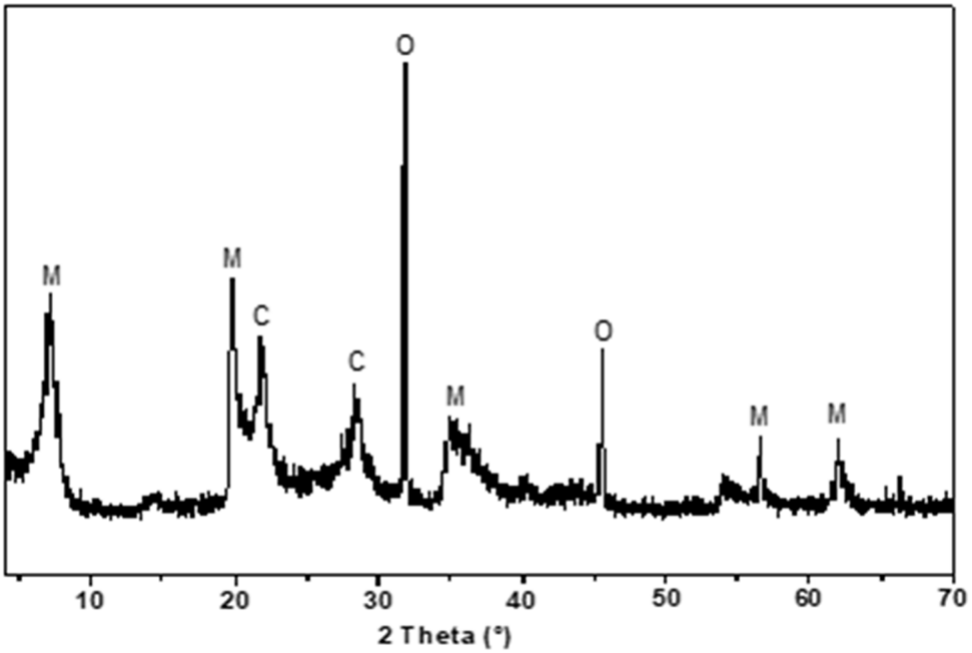
XRD of catalyst before to reuse. M: montmorillonite, C: cristobalite, and O: iron oxide.

To evaluate the recyclability of the catalyst, it was separated from the reaction mixture by centrifugation. The catalyst was then washed successively with methanol, ethyl acetate, and hexane to remove any residual product. Before reuse, the structure of the catalyst was confirmed by XRD. Fig. 15 shows the plane reflections observed in the diffractograms at (1 1 1), (2 0 2), and (2 2 0), which correspond to the same reflections as those the original catalyst, indicating that the catalyst's structure remained unchanged after recycling.

**Fig. 16:**
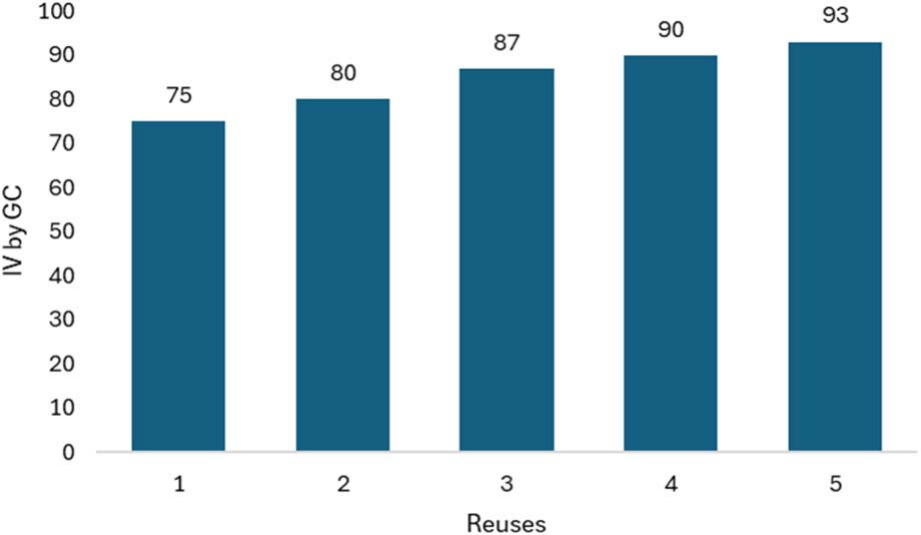
Decrease in IV with respect to time.

Since our objective is to establish a hydrogenation process that allows us to achieve IV values close to 70, we tested the reuse of the catalyst by monitoring changes in IV values after 60 minutes of reaction. The hydrogenation reactions were carried out at 550 psi of H_2_, using the Bent-Pd-2 catalyst at 70 °C. Fig. 16 shows the results over a range of one to five cycles of hydrogenation. While the efficiency of the catalyst gradually decreased after two runs, the IV values obtained increased until it ranched 93. This significant decrease is probably due to the presence of organic material at the catalytic sites, which cannot be completely removed despite washing with organic solvents.

#### 3.4.8 Hydrogenation on various supported metal catalysts

The efficiency of the Pd/Bentonite catalysts was compared with results previously reported in the literature for the hydrogenation of vegetable oils in the presence of other metal transition catalysts and supports (Table 9). Since an IV close to 70 is an important factor in quality control^43,93^ in the chemistry industry, we selected heterogeneous catalysts from the literature that report IV values near to 70 for this comparison. The values obtained for our catalyst were comparable to those obtained with Pd, Ni, and Pt-based catalysts. As shown in Table 9, similar IV values were obtained with these catalysts. The main differences lie in the pressure and temperature conditions used during the hydrogenation reaction. In this study, we achieved an IV value of 71 at 550 psi, which contrasts with the lower pressures used with the other catalysts (Table 9). However, our catalytic process was carried out at 70 °C, a lower temperature than those reported previously (Table 9).

**Table 9:** Comparison of the present work to other works obtained from recent literature

Catalyst	Metal loading (wt%)	Temperature and pressure	Initial IV	Final IV	Reaction time (min)	*Trans*-fats (%)	Saturated fats (%)	Oil feed employed	Ref.
Pd/SBA-15	Pd (1.00%)	110 °C, 73.47 psi	130	76	60	34.0	10.0	Sunflower	92
Pd–Ru/SBA-15	Pd (0.80%)	110 °C, 73.47 psi	130	70	60	29.0	18.0	Sunflower	98
	Ru (0.20%)								
Pd/γ-Al_2_O_3_	Pd (0.78%)	100 °C, 59.97 psi	125	90	20	19.9	5.6	Sunflower	91
Ni–Al–Ce	Ni (0.03%)	180 °C, 304.57 psi	115	70	13	20.9	18.1	Canola	99
	Ce (0.67%)								
Pt–Ni/SiO_2_	Pt (1.56%)	170 °C, 43.51 psi	128	70	300	16.8	23.0	Sunflower	62
	Ni (0.95%)								
Ni/ZnO/Al_2_O_3_	Ni (22.00%)	180 °C, 72.52 psi	162	70	130	22.8	33.5	Sunflower	44
Ni–Mg/SiO_2_	Ni (8.79%)	180 °C, 72.52 psi	124	70	59	13.7	34.4	Sunflower	46
Pt/γ-Al_2_O_3_	Pd (1.00%)	70 °C, 72.52 psi	133	82	65	3.5	27.8	Sunflower	61
Ni–Mg–Ag/diatomite	Ni (33.51%)	160 °C, 23.20 psi	130	90	255	26.3	5.8	Soybean	100
	Mg (1.39%)								
	Ag (5.88%)								
Ni–Cu–Mg/Kieselguhr	Ni (0.60%)	110 °C, 60.92 psi	130	70	75	21.1	20.6	Soybean	45
	Cu (0.22%)								
	Mg (0.17%)								
Ni (Pricat 9920)	Ni (21.00%)	110 °C, 73.47 psi	130	60	60	30.0	30.0	Sunflower	92
Pd/bentonite	Pd (1.60%)	70 °C, 550 psi	127	70	71	28.3	13.0	Soybean	This work
Pd/bentonite	Pd (1.60%)	25 °C, 550 psi	127	70	76	11.2	16.5	Soybean	This work

Table 9 also shows the metal loading used in each catalytic system. It is important to highlight that most systems reported in the literature, utilise a metal loading close to 1%, which is similar to our system, where the hydrogenation reaction was carried out with a Pd loading of 1.6%.

## 4. Conclusions

Partial hydrogenation of vegetable oil provides a valuable feedstock for chemical manufacturing and energy production. Recently, considerable efforts have focused on developing supported catalysts that enable the hydrogenation process to be carried out selectively under mild reaction conditions. We achieved this goal by developing a catalytic process that allowed the partial hydrogenation of soybean oil, reaching an IV value of 71 after 60 minutes at 70 °C, and an IV value of 76 after 120 minutes at 25 °C.

After 60 minutes at 70 °C, 28.33% TFAs and 13.01% saturated fatty acids were observed. In contrast, at 25 °C, TFAs decreased to 11.25%, while the saturated fatty acid content increased to 16.57%. The maximum concentration of TFA was observed at 70 °C, where TFA content reached a maximum of 30.49% after 80 minutes. At 25 °C, however, the maximum concentration of TFA was only 11.25%, and this was achieved after 120 minutes.

The Pd/bentonite catalyst, prepared by incipient wetness impregnation of Pd(ii) salt onto bentonite, exhibited a metal loading of 1.6%, which is comparable to that of Pd, Ni, and Pt-based catalysts. This catalyst offers several advantages, including its granular solid support, which facilitates easy filtration, and its ability to operate at 25 °C, contributing to energy savings.

## Data availability

All relevant data are included within the paper.

## Author contributions

JC and JAMS conceived the project and acquired the funds. SAFB, ET, RG, JC, and JAMS designed the experiments. SAFB, KHG, ET, RG, EGR, and OARM conducted the experimental work. RG, JC and JAMS coordinated the whole project. OARM, KHG, and JAMS wrote the manuscript. All the authors contributed to the discussions.

## Conflicts of interest

There are no conflicts to declare.
